# The spike protein of SARS-CoV-2 induces inflammation and EMT of lung epithelial cells and fibroblasts through the upregulation of GADD45A

**DOI:** 10.1515/med-2023-0779

**Published:** 2023-11-15

**Authors:** Jiehao Cai, Wenjie Ma, Xiangshi Wang, Hailing Chang, Zhongqiu Wei, Jingjing Li, Mei Zeng

**Affiliations:** Department of Infectious Diseases, Children’s Hospital of Fudan University, Shanghai 201102, China

**Keywords:** spike protein, SARS-CoV-2, lung cells, inflammation, EMT, GADD45A

## Abstract

Lung epithelial cells and fibroblasts poorly express angiotensin-converting enzyme 2, and the study aimed to investigate the role of the spike protein of severe acute respiratory syndrome coronavirus 2 (SARS-CoV-2) on inflammation and epithelial–mesenchymal transition (EMT) in two lung cell lines and to understand the potential mechanism. Lung epithelial cells (BEAS-2B) and fibroblasts (MRC-5) were treated with the spike protein, then inflammatory and EMT phenotypes were detected by enzyme-linked immunosorbent assay, Transwell, and western blot assays. RNA-sequence and bioinformatic analyses were performed to identify dysregulated genes. The roles of the candidate genes were further investigated. The results showed that treatment with 1,000 ng/mL of spike protein in two lung cell lines caused increased levels of IL-6, TNF-α, CXCL1, and CXCL3, and the occurrence of EMT. RNA-sequence identified 4,238 dysregulated genes in the spike group, and 18 candidate genes were involved in both inflammation- and EMT-related processes. GADD45A had the highest verified fold change (abs), and overexpression of GADD45A promoted the secretion of cytokines and EMT in the two lung cell lines. In conclusion, the spike protein induces inflammation and EMT in lung epithelial cells and fibroblasts by upregulating GADD45A, providing a new target to inhibit inflammation and EMT.

## 1 Introduction

Severe acute respiratory syndrome coronavirus 2 (SARS-CoV-2) has caused a pandemic of acute respiratory disease, named “coronavirus disease 2019” (COVID-19), with more than 240 million confirmed cases and 4.8 million deaths as of October 19, 2021 [1]. It has been reported that 81% of patients have mild disease with common symptoms of fever and cough, and 14% have severe disease requiring ventilation in an intensive care unit [2,3]. At the end of 2021, Molnupiravir and Nirmatrelvir-Ritonavir, two oral antiviral drugs, were approved for the treatment of patients with mild-to-moderate COVID-19 [4]. Before the approval and application of the drugs, vaccination and necessary personal preventive behaviors were still the most effective ways to prevent the transmission of SARS-CoV-2 [3].

Although most cases show mild symptoms when SARS-CoV-2 replicates in the lung epithelium in the early stages, some cases progress to complex symptoms, including respiratory dysfunction and systemic hyperinflammation [5]. Furthermore, multiple pieces of evidence indicate that prolonged COVID-19 can lead to lung fibrosis [6]. SARS-CoV-2 is a positive-sense single-stranded RNA virus that encodes four structural proteins: the envelope (E), nucleocapsid (N), spike (S), and membrane (M). SARS-CoV-2 has 79.5% similarity with SARS-CoV-1 at the genomic level [7]. One of the major differences is that 27 mutations are involved in the coding of the S protein [8], which is responsible for attachment to host cells in the initiation stage. This could explain the much higher affinity between angiotensin-converting enzyme 2 (ACE2) and S proteins [9,10] and the stronger infectivity [11] of SARS-CoV-2 than SARS-CoV-1. However, only a small subset of alveolar type II (AT2) cells express ACE2 [12], which is not expressed in other epithelial cells [13]. Therefore, inflammation and further fibrosis induced by SARS-CoV-2 or the S protein may be affected by non-AT2 cells independent of ACE2.

Notably, recent clues point out that the S protein is involved in the production of chemokines in non-AT2 cells in the initiation stage, which generally occurs in the amplification and consummation stages [14]. For instance, various inflammatory cytokines and chemokines have been detected in human lung epithelial cells stimulated with extracellular S protein [15]. Other studies also proved that this inflammatory reaction could be triggered by the S protein, independent of active viral infection and replication. Additionally, the effector cells include bronchial epithelial, microvascular endothelial, and airway epithelial cells [16,17]. Another study indicated that only the S protein, but not the E, N, or M proteins, can induce epithelial–mesenchymal transition (EMT) marker changes in breast cancer cells [18]. This could increase the risk of metastasis in cancer patients once they are infected. EMT is a complex process in which cells are transformed into mesenchymal cells through specific procedures, accompanied by a series of cytoskeletal and morphological changes, resulting in cell migration and invasion. This phenotypic alteration of cells is involved in cancer metastasis [19], chronic inflammation [19], chronic obstructive pulmonary disease, and lung fibrosis [20]. The effect of the S protein on the EMT of lung epithelial cells has not yet been investigated.

Lung fibroblasts are known to play pivotal roles in chronic respiratory disease and are involved in inflammation and lung fibrogenesis [21,22]. On the one hand, they are able to produce inflammatory cytokines [23]; on the other, they are regarded as effector cells through interactions with the injured alveolar epithelium in lung fibrogenesis [22]. Whether the inflammatory or migratory phenotype of lung fibroblasts can be directly induced or altered by the S protein is also unknown. These changes may be involved in the obvious symptoms or disease progression induced by SARS-CoV-2, including lung inflammation and fibrosis.

In the present study, we investigated the role of the S protein in inflammatory cytokine production and EMT in two non-AT2 cell lines – lung epithelial cells (BEAS-2B) and lung fibroblasts (MRC-5) – which poorly express ACE2 [13,24]. RNA-sequencing (RNA-seq) was performed to identify abnormally expressed genes, and bioinformatics analysis was used for the annotation of biological roles and screening of genes of interest. Finally, the functions of the candidate gene, growth arrest, and DNA damage-inducible gene alpha (GADD45A) in lung cells were investigated. Our study provides a new mechanism for understanding the role of S protein in lung inflammation and fibrosis induced by SARS-CoV-2.

## 2 Materials and methods

### 2.1 Cells and cell culture

Human lung epithelial cells (BEAS-2B) and human embryonic lung fibroblasts (MRC-5) were purchased from the Cell Bank of the Chinese Academy of Sciences (Shanghai, China). BEAS-2B cells were cultured in Dulbecco’s modified Eagle’s medium (DMEM, Gibco, Carlsbad, CA, USA) supplemented with 10% FBS and 1% penicillin/streptomycin. MRC-5 cells were cultured in minimum essential medium (MEM, Gibco) supplemented with the same supplements. All the cells were incubated at 37℃ with 5% CO_2_.

### 2.2 Cytotoxicity

The cells were seeded in 96-well plates at a density of 3,000 cells/well. Different concentrations (0, 10, 100, 500, 1,000, and 2,000 ng/mL) of recombinant S protein, purchased from ABclonal Technology Co., Ltd (Wuhan, China), were co-cultured in the wells for 3 days. The 3-(4,5-Dimethylthiazol-2-yl)-2,5-diphenyltetrazolium bromide (MTT) assay was used to detect the viability of the cells daily at the same time points. Briefly, the supernatant of wells was removed and 10 µL of 5 mg/mL MTT (Sigma-Aldrich; Merck KGaA, Darmstadt, Germany) mixed with the 90 µL of separate complete medium of each cell was added into each well. After incubation for 3 h, the unreacted MTT solution was removed carefully and 100 µL dimethyl sulfoxide was added to resolve the formazan, followed by incubation for another 30 min. The absorbance of each well was measured at 490 nm using a microplate reader. The relative cellular viability was used to determine cytotoxicity.

### 2.3 Enzyme linked immunosorbent assay (ELISA)

After the cells were treated with S protein (100 ng/mL) for 48 h, the supernatant was collected for ELISA. The production of inflammation cytokines (IL-6, TNF-α) and chemokines (CXCL1, CXCL3) were detected using the ELISA kits following the manufacturer’s instructions. The cytokine production levels were calculated using separate standard curves. Human IL-6 and TNF-α ELISA kits were purchased from ABclonal Technology Co., Ltd (Wuhan, China), and human CXCL1 and CXCL3 ELISA kits were provided by MULTISCIENCES (LIANKE) Biotech, Co., Ltd (Hangzhou, China).

### 2.4 Transwell assay

The cells were collected and resuspended in a separate medium without FBS supplementation at a density of 4 × 10^5^ cells/mL. Subsequently, 100 µL of the cell suspension was added to the center of the upper chamber of the Transwell insert (8 µm pore size; EMD Millipore, Billerica, MA, USA), and 600 µL of complete medium was added to the lower chamber. After 24 h of incubation, the upper chambers were collected and washed twice with PBS. Subsequently, the cells retained on the upper surface were removed, and those that migrated to the bottom surface of the chamber were fixed with methanol for 20 min and stained with 1% crystal violet for another 20 min. Finally, the migrated cells were photographed under a light microscope and five fields were randomly selected for cell counting.

### 2.5 Western blot

Total protein was extracted using a protein extraction kit (KeyGen, Jiangsu, China) and quantified using a BCA protein concentration detection kit (Beyotime, Shanghai, China) following the manufacturer’s instructions. Ten micrograms of protein were added to 12% SDS-PAGE gels for electrophoresis, and the separated proteins were transferred to polyvinylidene fluoride membranes. Next, 5% non-fat milk was added, and the membranes were incubated for 1 h. After washing thrice with tris buffered saline with tween-20, the membranes were cut and incubated with the diluted antibodies overnight, followed by incubation with the secondary antibody for 2 h. Subsequently, the signal of the desired protein was enhanced using an ECL kit (ABclonal) and captured on a gel imaging analysis system (Tanon, Shanghai, China). Primary antibodies against E-cadherin (1:1,000), Vimentin (1:1,000), N-cadherin (1:1,000), and GAPDH (1:1,000) were purchased from ABclonal. GAPDH was used as a loading control. The gray values of the bands were analyzed using the ImageJ software (National Institutes of Health, Bethesda, MD, USA).

### 2.6 RNA-seq

After cells were treated with S protein for 48 h, total RNA from each cell sample was collected using TRIzol reagent (Ambion, Thermo Fisher Scientific) and extracted using the RNeasy mini kit (Qiagen, Germany). Next, the RNA was sent to Sinotech Genomics Co., Ltd (Shanghai, China) for cDNA library construction. Briefly, mRNA molecules were first purified using poly T oligo-attached magnetic beads and then fragmented into small pieces for the synthesis of first- and second-strand cDNA. After an end repair, cDNA fragments was added with a single “A” base and ligated to the adapters. Finally, the cDNA was enriched by PCR to create a cDNA library and then sequenced on an Illumina NovaSeq 6000 (Illumina, USA). Dysregulated genes were screened using the following filtering criteria: fold-change (absolute) [FC(abs)] >2.

### 2.7 Construction of transcription factor (TF) regulatory network

TRANSFAC (http://genexplain.com/transfac) was used to predict the potential TFs of the differentially expressed genes (DEGs), and only TFs included in the identified DEGs were retained to explore their transcriptional regulatory roles. Finally, the TF regulatory network was visualized using Cytoscape software.

### 2.8 Bioinformatical analyses

Gene ontology (GO) and Kyoto Encyclopedia of Genes and Genomes (KEGG) signaling pathway analyses were performed to analyze the biological functions and potentially relevant signaling pathways of dysregulated genes. Gene numbers >2 and a *p* < 0.05 were used as thresholds to screen relevant GO terms and KEGG pathways. The terms and pathways were ranked in descending order according to the enrichment factor, and the top 30 terms and pathways were selected and presented in a separate bubble chart. The Venn diagram analysis was performed by Sinotech Genomics Co., Ltd (Shanghai, China).

### 2.9 qPCR

Total RNA was extracted using a universal RNA purification kit (TIANGEN, Beijing, China) following the manufacturer’s instructions and quantified using a microspectrophotometer (Allsheng, Hangzhou, China). RNA (500 ng) was reverse transcribed into cDNA using the PrimeScript RT Master Mix kit (TAKARA) according to the manufacturer’s protocol. Subsequently, 1 µL of cDNA was premixed with 0.5 µL of each primer, 10 µL of SYBR Green (TAKARA), and 8 µL of deionized water; the reaction system was transferred to a Real-time Thermal Cycler X960 (Heal-force, Shanghai, China) for amplification. The reaction was started at 95℃ for 5 min, followed by 40 cycles of 95℃ for 10 s and 60℃ for 60 s. Data were analyzed using the 2^−ΔΔCq^ method, and GAPDH was used as a normalization control. Primers were synthesized by Sangon Biotech Co., Ltd (Shanghai, China), and the sequences are listed in Table 1.

**Table 1:** Primer sequences used for qPCR

Gene name	sequences (5′–3′)	
	Forward	Reverse
FGF18	GATGGGGACAAGTATGCCCAG	GCCTTTGCGGTTCATGCAC
CHRM1	CTCTATACCACGTACCTGCTCA	CCGAGTCACGGAGAAGTAGC
MAPK15	GGGCCTATGGCATTGTGTG	TCTCTGGGCATCTGTCTTATCC
FGFR2	AGCACCATACTGGACCAACAC	GGCAGCGAAACTTGACAGTG
NODAL	CAGTACAACGCCTATCGCTGT	TGCATGGTTGGTCGGATGAAA
SERPINE1	ACCGCAACGTGGTTTTCTCA	TTGAATCCCATAGCTGCTTGAAT
ANGPT2	AACTTTCGGAAGAGCATGGAC	CGAGTCATCGTATTCGAGCGG
PTPRR	ACCTATCGCCCATCACATTACA	GCGGTGGTAGCTTTGATCTCA
IL24	TTGCCTGGGTTTTACCCTGC	AAGGCTTCCCACAGTTTCTGG
GADD45A	GAGAGCAGAAGACCGAAAGGA	CACAACACCACGTTATCGGG
GAPDH	CTGGGCTACACTGAGCACC	AAGTGGTCGTTGAGGGCAATG

### 2.10 Cell transfection

Cells were seeded in a six-well plate at a density of 3 × 10^5^ cells/mL, and the vectors containing full-length GADD45A were transfected into the cells using Lipofectamine ®2000 (Invitrogen; Thermo Fisher Scientific, Inc.) following the manufacturer’s instructions. Two days after transfection, the cells were collected for qPCR validation. The overexpression vector of GADD45A was synthetized by Sangon Biotech Co., Ltd (Shanghai, China), and an empty vector was used as a negative control (NC).

### 2.11 Statistical analysis

Data were presented as mean ± standard deviation, and each experiment was performed at least three times. All analyses were performed using SPSS software (version 16.0; SPSS, Inc.). Student’s *t*-test was used to evaluate the statistical significance of the differences between two groups. Statistical significance was set at *p* < 0.05.

## 3 Results

### 3.1 The S protein induced inflammatory molecule secretion and EMT of lung cells

**Figure 1:**
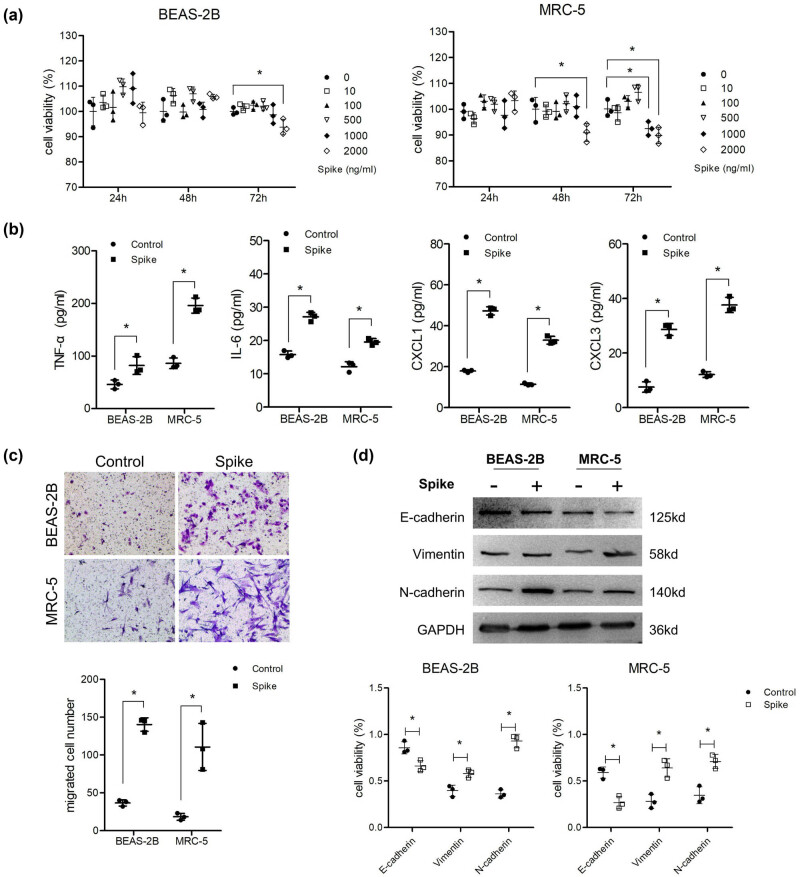
Spike protein induced inflammatory molecule secretion and EMT of lung cells. (a) Cells were co-cultured with different concentrations of S protein for 24, 48, and 72 h. MTT was used to detect cell viability (*n* = 3). After cells were treated with 1,000 ng/mL S protein for 48 h, (b) ELISA was used to analyze the supernatant levels of TNF-α, IL-6, CXCL1, and CXCL2 (*n* = 3). (c) Transwell assay was performed to detect migration ability (*n* = 3) and (d) western blotting was used to detect the protein levels of E-cadherin, vimentin, and N-cadherin (*n* = 3).

First, the cytotoxicity of the S protein was detected using the MTT assay. The results showed that the viability of the two cell lines treated with S protein was similar to that of the control group at a dose of 10–500 ng/mL at all treatment time points, and a small reduction in viability was observed in MRC-5 cells treated with 1,000 and 2,000 ng/mL for 48 or 72 h, respectively (Figure 1a). This indicated a slight cytotoxicity of the S protein, and the highest safe dose and treatment time (1,000 ng/mL and 48 h) were selected for the following assays. The production of inflammatory cytokines (IL-6, TNF-α) were increased by ∼1-fold, and the chemokines (CXCL1, CXCL3) were upregulated by 2–3-fold after cells were treated with S protein for 48 h through ELISA detection (Figure 1b). Transwell assay results showed that the migration abilities of both cell types were significantly enhanced compared to those of the control group (Figure 1c). EMT of cells also occurred with higher levels of vimentin and N-cadherin and lower levels of E-cadherin (Figure 1d).

### 3.2 Dysregulated genes identified by RNA-seq after S protein treatment

**Figure 2:**
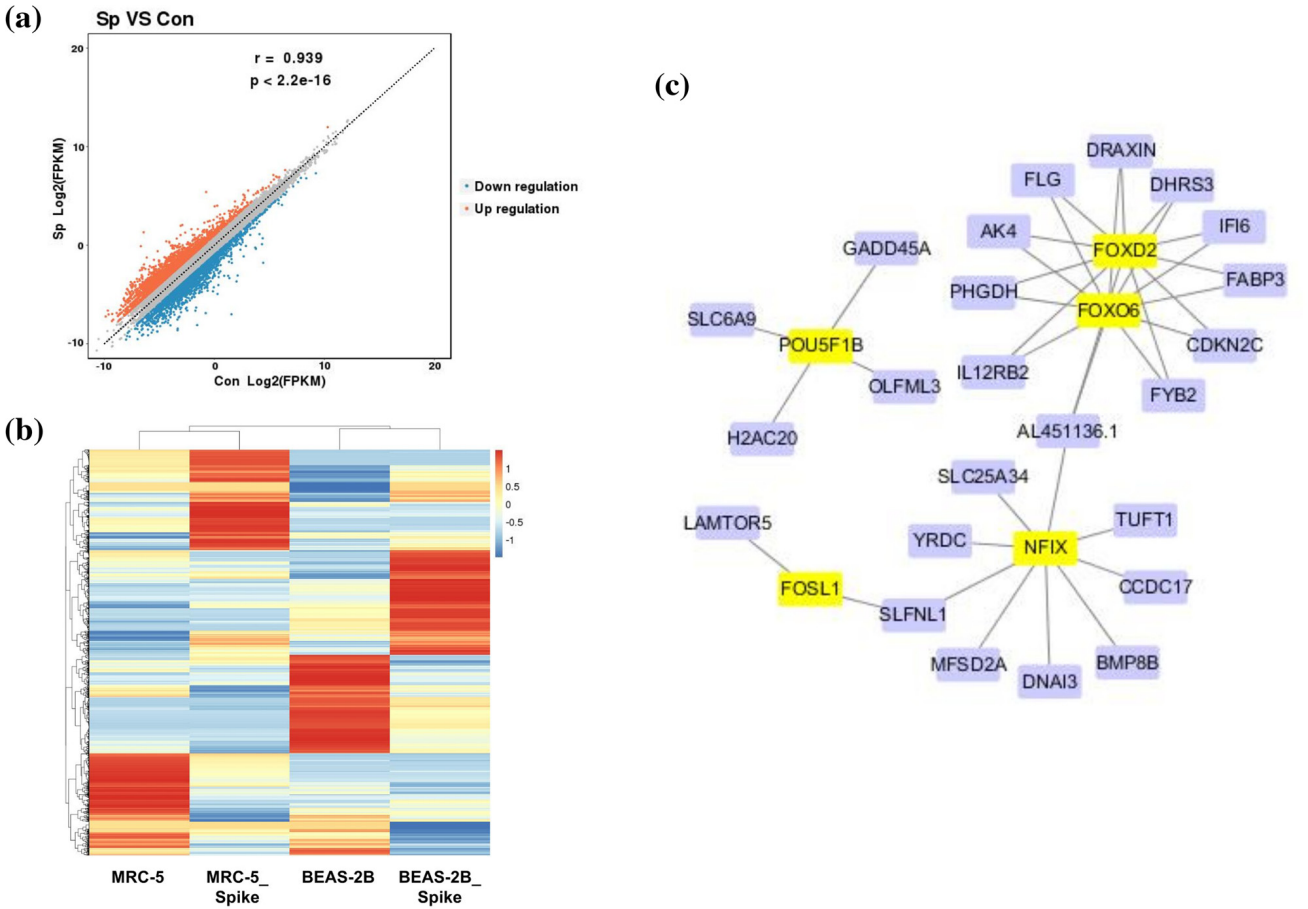
Dysregulated genes identified by RNA-seq after S protein treatment. RNA-seq was performed to identify the dysregulated genes after the cells were treated with 1,000 ng/mL S protein for 48 h (*n* = 1). (a) Scatter plot of dysregulated genes. Sp, spike group; Con: Control group. (b) Heat map of dysregulated genes. Each line indicates a gene and each column indicates a cell sample. (c) TF regulatory network constructed from dysregulated genes. Purple rounded rectangles, genes; yellow rounded rectangles, TF.

After the cells were treated with S protein for 48 h, RNA-seq was used to identify abnormally expressed genes. As shown in Figure 2a, 4,238 dysregulated genes were screened (including 2,144 upregulated and 2,094 downregulated genes) under the threshold of [FC(abs)] >2. The heat map in Figure 2b indicates that the expression profiles of the four samples varied significantly, and several clusters of gene expression trends between the two different samples in the spike group were consistent. Table 2 lists the alterations in the expression of the top ten genes with the highest FC (abs) values, which varied from approximately 40 to 400. Furthermore, a TF regulatory network of all dysregulated genes was constructed to uncover the regulatory relationships between genes. Only a small number of genes were identified, including five TFs (POU5F1B, FOXO6, FOXD2, FOSL1, and NFIX) and 24 target genes (Figure 2c). Two TFs (FOXO6 and FOXD2) shared 11 target genes.

**Table 2:** Top ten dysregulated genes after Spike protein treatment

	Gene id	Gene name	log2FC	log2FC abs	FC abs	Up/down
1	ENSG00000283515	AC020915.4	−8.57	8.57	379.60	Down
2	ENSG00000274049	INO80B-WBP1	7.74	7.74	213.32	Up
3	ENSG00000068781	STON1-GTF2A1L	6.58	6.58	95.48	Up
4	ENSG00000285238	AC006064.6	−6.55	6.55	93.69	Down
5	ENSG00000244563	RPS26P19	−6.23	6.23	74.81	Down
6	ENSG00000275121	AC211486.4	6.15	6.15	70.78	Up
7	ENSG00000248405	PRR5-ARHGAP8	−6.11	6.11	68.90	Down
8	ENSG00000242419	PCDHGC4	−5.70	5.70	51.83	Down
9	ENSG00000127954	STEAP4	−5.29	5.29	39.06	Down
10	ENSG00000103426	CORO7-PAM16	−5.24	5.24	37.78	Down

### 3.3 Screen of candidate genes regulating inflammation and EMT following GO and KEGG analyses

**Figure 3:**
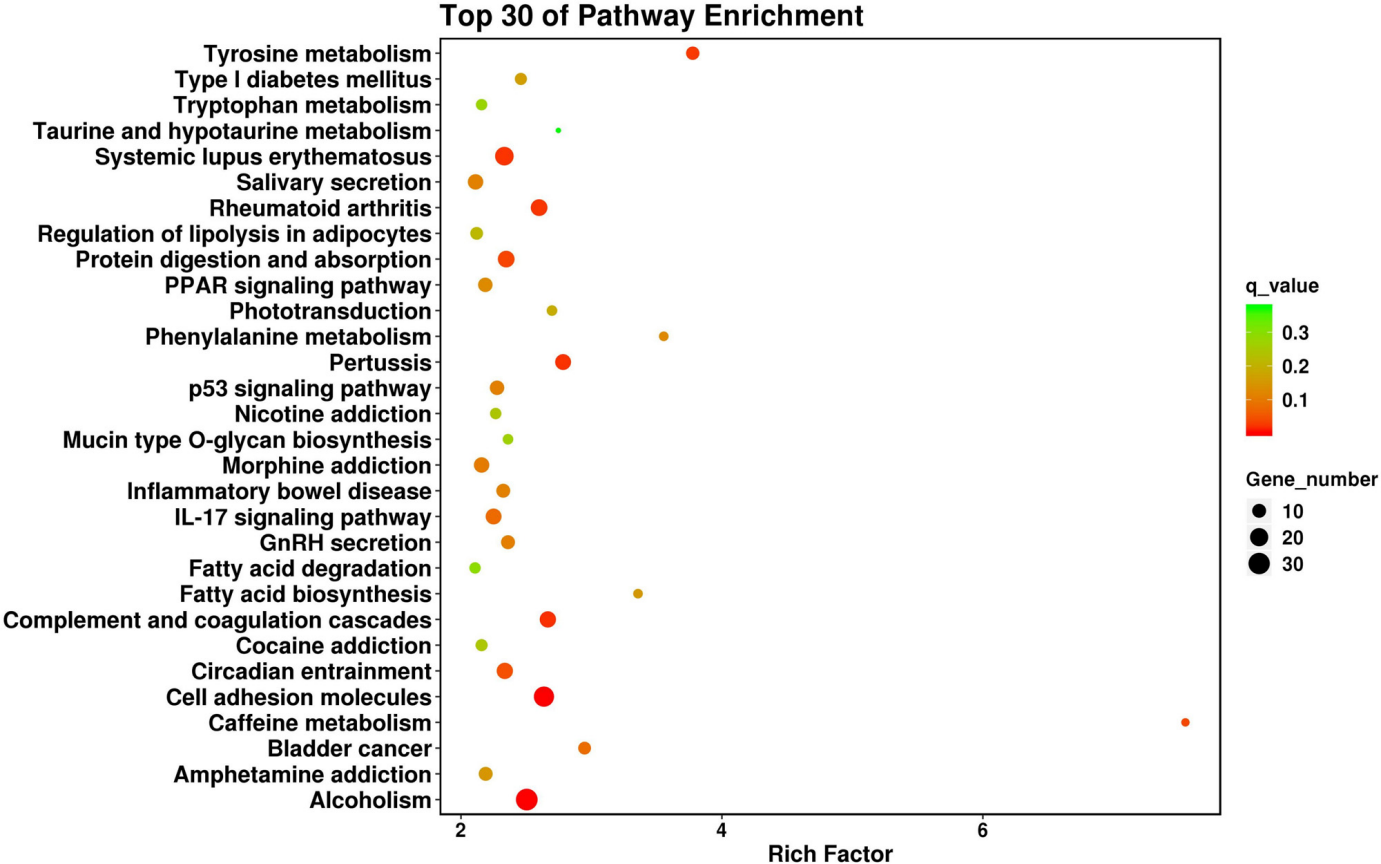
Top 30 of KEGG pathway enrichment analysis of the dysregulated genes.

GO analysis indicated that the dysregulated genes were mainly enriched in several terms, including G protein-coupled acetylcholine receptor activity, water transmembrane transporter activity, and the complement receptor-mediated signaling pathway (Table S1). KEGG pathway enrichment analysis showed that the genes were mainly involved in rheumatoid arthritis, inflammatory bowel disease, and p53, IL-17, and PPAR signaling pathways (Figure 3).

**Figure 4:**
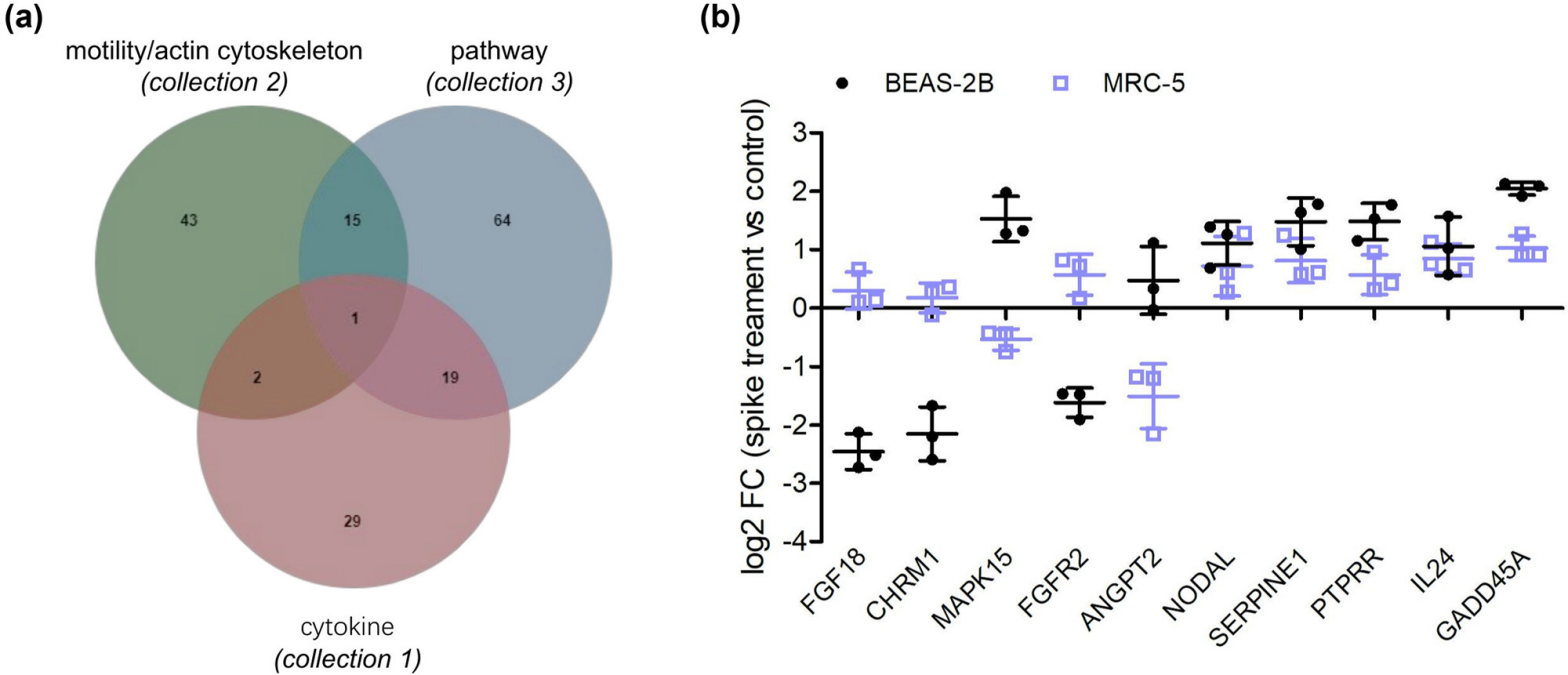
Screen and validation of candidate genes. (a) Venn diagram analysis of overlapped genes screened between collection 2 and collection 1 or 3. (b) qPCR validation of the fold changes of the top ten overlapped genes in two lung cells treated with 1,000 ng/mL S protein for 48 h (*n* = 3).

It was relatively difficult to screen candidates for the altered phenotype of cells following analysis of the top 30 terms or pathways. Therefore, several keywords (“cytokine,” “motility,” and “actin cytoskeleton”) were used to screen relevant terms and pathways in Tables S1 and S2. As a result, two terms (GO:0004896 and GO:0005125) and two pathways (hsa04060 and hsa04061) were screened out under the threshold of *p* < 0.05 (Table 3). Many inflammatory cytokines (IL24, IL20, and IL1A) and chemokines (CXCL1, CXCL6, CXCL3, and CXCL5) were upregulated, which is consistent with our ELISA results. In addition, 61 genes were enriched in the negative regulation of cell motility and regulation of the actin cytoskeleton; these genes may play important roles in the regulation of migration ability and EMT. Venn diagram analysis indicated that three genes (IL24, NODAL, and HMGB1) were involved in both collection 1 (cytokines) and collection 2 (motility/actin cytoskeleton) (Figure 4a). Next, eight pathways involved in the signal transduction of SARS-CoV-2 infection were selected from Table S2, and a total of 18 overlapping genes were obtained by Venn diagram analysis of collections 2 and 3 (pathway). The screened genes are listed in Table 4 according to the FC(abs) values.

**Table 3:** Genes enriched in cytokine- and migration-related biological processes and pathways

Keywords (collection)	GO/pathway_ID	GO term/pathway_DES	Up_ genes	Down_ genes	Gene_up_list	Gene_down_list
Cytokine (collection 1)	hsa04060	Cytokine-cytokine receptor interaction	28	12	EDA2R, IL13RA2, CXCL1, BMP2, GDF15, IL24, FAS, IL23R, IL1A, NODAL, INHBB, IL20, IL12RB2, CXCL6, TNFSF15, CCR1, TNFRSF10C, TNFRSF9, MSTN, CSF2RB, CXCL8, CXCL3, TNFSF13B, IL9R, CXCL5, IL1RL1, IL31RA, GDF1	CNTFR, IL1R2, TNFRSF8, TNFSF10, EDAR, BMP8B, IL13, BMP6, IL34, INHBE, CCR10, CXCL16
	hsa04061	Viral protein interaction with cytokine and cytokine receptor	9	3	CXCL1, IL24, IL20, CXCL6, CCR1, TNFRSF10C, CXCL8, CXCL3, CXCL5	TNFSF10, IL34, CCR10
	GO:0004896	Cytokine receptor activity	10	5	IL1RL1, IL12RB2, IL13RA2, IL31RA, CSF2RB, IL17RD, IL9R, IL23R, CCRL2, CCR1	CNTFR, GFRA3, GPR17, IL1R2, CCR10
	GO:0005125	Cytokine activity	20	10	CXCL8, CMTM2, TNFSF15, CXCL5, BMP2, GDF15, CXCL6, CXCL3, WNT5B, MSTN, NODAL, NDP, IL20, IL24, SPP1, IL1A, GDF1, INHBB, CXCL1, TNFSF13B	INHBE, HMGB1, BMP6, IL34, AREG, CXCL16, BMP8B, IL13, TNFSF10, WNT8B
Motility/actin cytoskeleton (collection 2)	GO:2000146	Negative regulation of cell motility	18	23	TIE1, MIR503, MIR221, MIR10A, STC1, MIR424, NODAL, NAV3, SLIT2, SERPINE1, DDT, IL24, TMIGD3, PTPRR, GADD45A, ARHGDIB, DNAI3, HAS1	CDH1, MEOX2, HMGB1,GPR18, CHRD, LDLRAD4, MIR149, TMEFF2, ANGPT2,CLDN5, SERPINF1, MARVELD3, KRT16, IFITM1, MIR199A1, MYOCD, PADI2, SPOCK3, SVBP, MAPK15, MIR29B2, TACSTD2, C5AR2
	hsa04810	Regulation of actin cytoskeleton	8	12	SCIN, CYFIP2, PIP5K1B, ITGAX, EGF, CHRM5, PAK6, PIK3R1	ACTR3C, FGFR3, FGF18, PDGFD, FGF22, PPP1R12B, CHRM4, IQGAP2, CHRM1, FGF7, FGFR2, LPAR5
Pathway (collection 3)	hsa03320	PPAR signaling pathway	4	7	MMP1, SLC27A6, ACSL4, ACSBG,	LPL, FABP7, FABP3, ACSBG2, FABP4, PLIN1, AQP7
	hsa04115	p53 signaling pathway	11	10	CCND1, SESN2, SERPINE1, FAS, MDM2, SERPINB5, GADD45A, CCND2, ZMAT3, TP53I3	RRM2
	hsa04657	IL-17 signaling pathway	14	10	CXCL1, MMP3, CXCL6, FOSL1, MMP1, FOSB, PTGS2, CXCL8, CXCL3, CXCL5	MUC5B, IL13, JUN, MAPK15
	hsa04010	MAPK signaling pathway	11	22	DUSP2, HSPA2, DUSP4, FAS, IL1A, TGFA, GADD45A, FLT4, PTPRR, EGF, DUSP16	FGFR3, FGF18, FLT1, RASGRP2, PDGFD, FGF22, ANGPT2, CACNA1I, RASGRP3, EFNA3, DUSP9, NFATC1, CACNG6, EFNA2, AREG, IGF2, EFNA1, CACNG7, JUN, FGF7, CACNA2D3, FGFR2
	hsa04151	PI3K-Akt signaling pathway	16	22	TLR4, CCND1, VWF, LAMC2, MDM2, TGFA, GNG13, GNG2, FLT4, EGF, CCND2, SPP1, PIK3R1, LAMB3, C8orf44-SGK3, GNG8	FGFR3, DDIT4, FGF18, FLT1, LPAR6, TNXB, PDGFD, FGF22, ANGPT2, GNG7, GNG4, EFNA3, COL4A3, EFNA2, AREG, IGF2, EFNA1, CHRM1, FGF7, FGFR2, LPAR5, VTN
	hsa04668	TNF signaling pathway	8	3	CXCL1, FAS, MMP3, CXCL6, PTGS2, PIK3R1, CXCL3, CXCL5	RIPK3, SOCS3, JUN
	hsa04064	NF-κB signaling pathway	9	1	TLR4, EDA2R, CXCL1, GADD45A, PTGS2, CXCL8, CXCL3, TNFSF13B, LBP	EDAR
	hsa04630	JAK-STAT signaling pathway	12	3	IL13RA2, CCND1, IL24, IL23R, IL20, IL12RB2, EGF, SOCS7, CCND2, CSF2RB, PIK3R1, ENSIL9R	CNTFR, IL13, SOCS3

**Table 4:** Eighteen overlapped genes screened between collection 2 and collection 1 or 3

	Gene id	Gene name	log2FC	log2FC abs	FC abs	Up/down
1	ENSG00000156427	FGF18	−3.17	3.17	9.01	Down
2	ENSG00000168539	CHRM1	−2.81	2.81	7.01	Down
3	ENSG00000181085	MAPK15	−1.95	1.95	3.86	Down
4	ENSG00000066468	FGFR2	−1.85	1.85	3.62	Down
5	ENSG00000156574	NODAL	1.81	1.81	3.49	Up
6	ENSG00000106366	SERPINE1	1.76	1.76	3.39	Up
7	ENSG00000091879	ANGPT2	−1.65	1.65	3.13	Down
8	ENSG00000184574	LPAR5	−1.50	1.50	2.84	Down
9	ENSG00000153233	PTPRR	1.46	1.46	2.75	Up
10	ENSG00000162892	IL24	1.38	1.38	2.61	Up
11	ENSG00000116717	GADD45A	1.31	1.31	2.49	Up
12	ENSG00000138798	EGF	1.25	1.25	2.38	Up
13	ENSG00000170962	PDGFD	−1.25	1.25	2.37	Down
14	ENSG00000070388	FGF22	−1.22	1.22	2.34	Down
15	ENSG00000145675	PIK3R1	1.18	1.18	2.27	Up
16	ENSG00000068078	FGFR3	−1.17	1.17	2.25	Down
17	ENSG00000189403	HMGB1	−1.15	1.15	2.23	Down
18	ENSG00000140285	FGF7	−1.14	1.14	2.20	Down

### 3.4 Overexpression of GADD45A promoted inflammatory molecule secretion and EMT of lung cells

**Figure 5:**
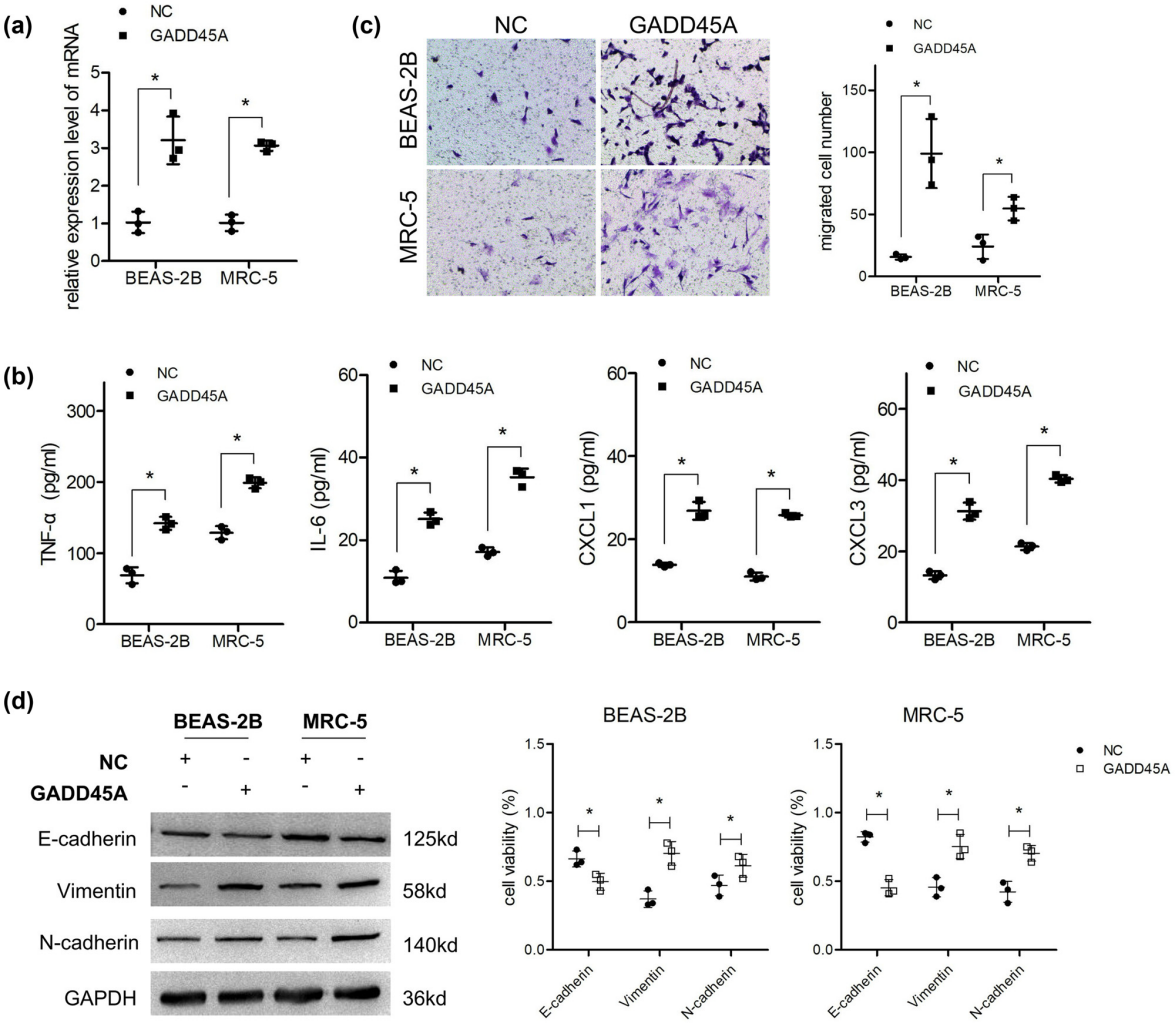
Overexpression of GADD45A promoted inflammatory molecule secretion and EMT of lung cells. (a) Cells were transfected with GADD45A overexpression vector, and qPCR was performed to detect the transfection effect (*n* = 3). After cells overexpressed with GADD45A, (b) ELISA was used to analyze the supernatant levels of TNF-α, IL-6, CXCL1, and CXCL2 (*n* = 3). (c) Transwell assay was performed to detect migration ability (*n* = 3) and (d) western blotting was used to detect the protein levels of E-cadherin, vimentin, and N-cadherin (*n* = 3).

The top five upregulated and five downregulated candidates were selected for qPCR validation. The results in Figure 4b show that the log2FC values of some genes (GFG18, CHRM1, and MAPK15) in MRC-5 were very low (nearly 0), and that the values of FGFR2 and ANGPT2 were not consistent between the two cell lines; however, the remaining five genes increased. Among the five upregulated genes, GADD45A had the highest validated FC (abs) in both cell lines (Figure 4b). Next, the overexpression vector of GADD45A was transfected into lung cells and confirmed by qPCR (Figure 5a). ELISA results indicated that lung cells overexpressing GADD45A secreted higher levels of inflammatory molecules than those in the NC group (Figure 5b). Furthermore, the migration ability of the two lung cell lines was enhanced after the overexpression of GADD45A (Figure 5c). Consistently, GADD45A induced significant EMT in lung cells (Figure 5d).

## 4 Discussion

Lung inflammation is the most common symptom of SARS-CoV-2, and fibrosis occurs in some COVID-19 cases [6]. Mutation of the S protein of SARS-CoV-2 [8] and the inflammation and EMT phenotype triggered by the S protein (but not other structural proteins) [15,18] indicate the potential complex roles of the S protein in the progression of relevant symptoms and diseases after infection. Because the expression level of ACE2 in AT2 cells is very limited, let alone in other lung cells [12,13], focusing on non-AT2 cells is helpful in understanding and determining the changes that occur in the early stages of infection. To date, inflammation triggered by a single S protein has been confirmed in macrophages and epithelial cells [16,25,26]. On the other hand, lung fibroblasts – the major cells involved in chronic respiratory disease and lung fibrogenesis – are often neglected. Additionally, the EMT of lung epithelial cells or fibroblasts induced by the S protein remains unknown. Therefore, in the present study, the role of the S protein in fibroblasts (MRC-5) was also investigated to uncover the potential shared mechanism of inflammation and EMT induced by the S protein in both lung epithelial cells and fibroblasts.

In contrast to macrophages, the production of inflammatory molecules stimulated by S protein in epithelial cells is much slower, often requiring 12 or 24 h [15]. In the present study, 48 h was selected as the stimulation time for the two cell types for both phenotype and RNA-seq analyses. Our result indicated that S protein treatment significantly increased proinflammatory molecule expression (IL-6, TNF-α, CXCL1, CXCL3) from the two cells. In addition, EMT of the two lung cell lines was detected after S protein stimulation. EMT could be triggered by multiple pathways including Wnt, nuclear factor (NF)-kappa B (κB), and transforming growth factor β pathways. Additionally, NF-κB activated by inflammatory cytokines are thought to further accentuate EMT in the lung [20]. Moreover, cells undergoing partial EMT exhibit enhanced inflammation [27]. This suggests that the S protein triggers EMT and inflammation in lung cells, resulting in consistently increased levels of EMT and inflammation. Once proinflammatory cytokines and chemokines enter the bloodstream, a number of immune cells are recruited to initiate a defense mechanism, which may promote a cytokine storm [28].

To investigate the potential mechanism, RNA-seq was performed to screen for abnormally expressed genes in lung cells. As expected, the gene expression profiles of the two cell types differed significantly. Fortunately, several clusters of gene expression trends were consistent after S protein treatment in the absence of a *p*-value. Although the top ten genes showed a significant fold change, clues supporting their roles in inflammation or EMT were less definite, according to the bioinformatics analysis results. Therefore, GO terms and KEGG pathways related to inflammation and EMT were directly screened and several candidates were obtained through overlapping analysis. Recent studies have indicated that multiple pathways are involved in SARS-CoV-2 infection. The NF-κB pathway is a classical pathway regulating inflammation, and the activation of NF-κB has been confirmed in various cell types from COVID-19 patients [29] or post-stimulation with S protein [30] and its subunit S1 [31]. Activation of the p38 mitogen-activated protein kinase (MAPK) signaling pathway is considered one of the major pathways involved in many viral infections, and treatment with p38 or MEK inhibitors effectively inhibits the infectivity of SARS-CoV-2 and its S protein [32,33]. Furthermore, the PPAR [34], PI3K/AKT [16], and p53 [35] signaling pathways were all activated in SARS-CoV-2 and S protein-infected samples. Therefore, an overlapping analysis of genes enriched in these pathways and EMT-related biological processes is important for screening candidate genes.

GADD45A belongs to the GADD45 family and is transcriptionally activated by various stress stimuli. The protein encoded by GADD45A is small (18 kDa) and plays an important role in regulating DNA repair, cell proliferation, survival, differentiation, and immune responses by interacting with several intracellular signaling molecules [36,37]. Recent studies have indicated the complex roles of GADD45A in inflammation. Mathew et al. found that lung GADD45A was significantly enhanced after a single-dose thoracic radiation, and mice deficient in GADD45A showed increased susceptibility to radiation-induced lung injury and higher levels of inflammatory cytokines [38]. This indicates that GADD45A is an important modulator of lung inflammatory responses. GADD45b can also be induced in T cells by the pro-inflammatory cytokines IL-12 and IL-18 [39]. Additionally, studies have uncovered the important role of GADD45A in regulating migration by functioning as a target of miRNAs [40,41]. These findings reflect the dual roles of GADD45A in regulating inflammation and EMT, which are consistent with the results of the overlapping analysis and phenotype verification assay.

In the present study, GADD45A was upregulated after lung cells were treated with S protein, which can be sorted as an environmental stimulus. The p38/MAPK pathway is considered the major activated pathway when GADD45A responds to stimuli [36], and it could be the upstream signal transduction pathway for increased GADD45A. We also noted that GADD45A is one of the target genes of the TF POU class 5 homeobox 1 B (POU5F1B), which plays important roles in carcinogenesis [42,43]. Whether abnormal expression of this TF contributes to the upregulation of GADD45A requires further investigation. A previous study indicated that proinflammatory cytokines induce the expression of GADD45A [39]; hence, we proposed that autocrine inflammatory molecules (induced by the S protein) could further promote the expression of GADD45A.

Taken together, our study indicates that the S protein is able to induce inflammation and EMT in lung epithelial cells and fibroblasts through the upregulation of GADD45A, which provides a new target to inhibit inflammation and relevant fibrosis. More importantly, our study showed that lung fibroblasts are another group of effector cells of the S protein, providing new target cells to combat the virus. The gene expression profiles of lung cells in response to the S protein also provided a number of candidate genes for a better understanding of the mechanism of the S protein.

## Supplementary Material

Supplementary Table S1

Supplementary Table S2
